# Influence of Menstruation upon the Exanthemata

**Published:** 1846-05

**Authors:** 


*Influence of Menstruation upon the Exanthemata.*—The appearance of the catamenia, in the course of any of the acute exanthemata, is always to be regarded as an unpleasant symptom. Cases, indeed, occur, where the act of menstruation seems to have a truly critical salutary influence upon the existing fever; the violent pyrexial and the grave nervous symptoms subsiding immediately upon its development. But the very opposite state of things is much more common; and most experienced physicians must have met with cases where the occurrence of this secretion, during the eruptive stage of Measles, for example, has been speedily followed by alarming oppression and anxiety, and perhaps by death. If the case does not prove fatal, the eruption will generally be observed to become more or less livid, and to have petechial spots mixed with it; these spots may be distinguished from the proper exanthem, by not disappearing under the pressure of the finger. A not unfrequent symptom in such circumstances—and one of the worst augury—is a very fetid odor of the breath, the tongue being at the same time parched, and covered with a dry brown crust. Again, when the catamenia are entirely suppressed, the prognosis must be extrmely unfavorable. It is therefore obvious that the state of this function must influence very materially our opinion as to the danger of exanthematous fevers in menstruating females. Many of the older writers have alluded to the very unfavorable change that is apt to occur in the eruption of small-pox, by the occurrence of menstruation. The pustules are apt to become livid from the admixture of a sanguinolent fluid with their contents, and the large patches of ecchymosis to make their appearance on different parts of the surface. The danger is the greater, if the catamenia occur upon the seventh or eleventh day of the exanthem.* An inexpressible oppression or anxiety is then apt to come over the patient; she feels inwardly assured (a genuine *clairvoyane*) of her approaching dissolution; her pulse becomes exceedingly weak and rapid; her feet cold; and in a few hours, perhaps, death will close the scene, although everything had, shortly before, seemed propitious and favorable.

Dissection in these cases usually discovers an unusual fluidity of the blood, and—a common consequence of this morbid state—a dark-red coloring of the inner surface of the bloodvessels; such as we frequently observe in cases of death from lightning. This appearance has been erroneously attributed to inflammation; whereas it is the mere result of imbibition or Endosmosis of the vascular parietes.—*Ibid.*

* In a case of Scarlatina, related in these reports, Schoenlein advises medical men never to give a confident favorable prognosis in this disease, until the eleventh day is past.

